# Enterovirus 71 Activates GADD34 via Precursor 3CD to Promote IRES-Mediated Viral Translation

**DOI:** 10.1128/spectrum.01388-21

**Published:** 2022-01-05

**Authors:** Hui Li, Wenqian Li, Shuangling Zhang, Manman Qiu, Zhuoran Li, Yongquan Lin, Juan Tan, Wentao Qiao

**Affiliations:** a Key Laboratory of Molecular Microbiology and Technology, Ministry of Education, College of Life Sciences, Nankai University, Tianjin, China; Wright State University

**Keywords:** GADD34, EV71, non-structural precursor 3CD, IRES-mediated translation, enterovirus, virus-host interactions

## Abstract

Enterovirus 71 (EV71) is the major pathogen of hand, foot, and mouth disease. In severe cases, it can cause life-threatening neurological complications, such as aseptic meningitis and polio-like paralysis. There are no specific antiviral treatments for EV71 infections. In a previous study, the host protein growth arrest and DNA damage-inducible protein 34 (GADD34) expression was upregulated during EV71 infection determined by ribosome profiling and RNA-sequencing. Here, we investigated the interactions of host protein GADD34 and EV71 during infections. Rhabdomyosarcoma (RD) cells were infected with EV71 resulting in a significant increase in expression of GADD34 mRNA and protein. Through screening of EV71 protein we determined that the non-structural precursor protein 3CD is responsible for upregulating GADD34. EV71 3CD increased the RNA and protein levels of GADD34, while the 3CD mutant Y441S could not. 3CD upregulated GADD34 translation via the upstream open reading frame (uORF) of *GADD34* 5'untranslated regions (UTR). EV71 replication was attenuated by the knockdown of GADD34. The function of GADD34 to dephosphorylate eIF2α was unrelated to the upregulation of EV71 replication, but the PEST 1, 2, and 3 regions of GADD34 were required. GADD34 promoted the EV71 internal ribosome entry site (IRES) activity through the PEST repeats and affected several other viruses. Finally, GADD34 amino acids 563 to 565 interacted with 3CD, assisting GADD34 to target the EV71 IRES. Our research reveals a new mechanism by which GADD34 promotes viral IRES and how the EV71 non-structural precursor protein 3CD regulates host protein expression to support viral replication.

**IMPORTANCE** Identification of host factors involved in viral replication is an important approach in discovering viral pathogenic mechanisms and identifying potential therapeutic targets. Previously, we screened host proteins that were upregulated by EV71 infection. Here, we report the interaction between the upregulated host protein GADD34 and EV71. EV71 non-structural precursor protein 3CD activates the RNA and protein expression of GADD34. Our study reveals that 3CD regulates the uORF of the 5′-UTR to increase GADD34 translation, providing a new explanation for how viral proteins regulate host protein expression. GADD34 is important for EV71 replication, and the key functional domains of GADD34 that promote EV71 are PEST 1, 2, and 3 regions. We report that GADD34 promotes viral IRES for the first time and this process is independent of its eIF2α phosphatase activity.

## INTRODUCTION

Enterovirus 71 (EV71) belongs to the *Enterovirus* genus of the *Picornaviridae* family and is also the major pathogen of hand, foot, and mouth disease (HFMD) in infants and children. EV71 infection causes fever, pharyngitis, oral ulcers, and rashes on the hands and feet. In severe cases, it can cause neurological complications and even life-threatening disease. EV71 has broken out and spread in many parts of the world and emerged as a serious public health concern. The EV71 genome is a single positive-strand RNA, about 7,400 nt in length. One open reading frame (ORF) encodes a polyprotein precursor which cleaves into four structural proteins (VP1-4) and seven non-structural proteins (2A-C and 3A-D). Several non-structural precursor proteins that are not completely cleaved, such as 2BC, 3AB, and 3CD, have also been reported to perform important functions (1, 2). The virus shifts the intracellular resources toward virus replication by host shutoff (3). The 2A protease cleaves the eukaryotic translation initiation factor 4G (eIF4G) and shuts down the translation of host genes (4). The 3C protease enters the nucleus in the form of the precursor 3CD, cleaving various transcription factors and poly(A) binding protein (PABP), and inhibiting cellular transcription and translation (5, 6). EV71 genomic RNA 5′-untranslated regions (UTR) contains two parts of functional elements important for translation and replication: cloverleaf (stem-loop I) and internal ribosome entry site (IRES, stem-loop II to VI). The cloverleaf structure is related to viral RNA replication, and the IRES is responsible for the initiation of cap-independent translation of viral proteins (7). Various host proteins have been identified to regulate the IRES activity, known as IRES *trans*-acting factors (ITAFs) (8). Currently, the identification and mechanism of action of ITAFs is still being expanded.

3CD is a multifunctional precursor protein produced by the major processing pathway of picornavirus. 3CD has protease and RNA binding activity, but its specificity is different from 3C (9). 3CD does not show any RNA-dependent RNA polymerase (RdRp) activity like 3D. 3CD has a nuclear localization signal (NLS) and localizes to the nucleus during infection (10). In addition, 3CD is also involved in the formation of replication organelles (ROs) and the assembly of virus particles (11). Recently, the most compelling discussions on 3CD functions revolve around 3CD to relocate Arf1 and to regulate membrane transport (11). Plus-strand RNA virus remodels the intracellular membrane to form ROs, conducive to immune escape or creating an environment suitable for replication. ADP ribosylation factors (Arfs) are the focus of regulating membrane transport exploited by viruses. Different from the lipid composition of general cell membranes, phosphatidylinositol-4-phosphate (PI4P) is usually a marker of picornavirus ROs (2). 3CD regulates the three guanine-nucleotide exchange factors (GEF) of Arf1, including Golgi-specific brefeldin A-resistance GEF (GBF1), BIG1, and BIG2, thereby activating Arf1 by replacing GDP with GTP (2, 12). 3CD induces PI4P synthesis by activating Arf1 and may regulate cellular phospholipid and membrane biogenesis by hijacking cellular pathways (2). The four single point mutations of PV 3CD, F441S, K12L, L630D, and R639D can eliminate the activation of Arf (2, 12, 13). RNA viruses usually have a small genome and a high mutation rate. Viral proteins often perform multiple roles to make full use of the genome capacity. 3CD has different functions from 3C and 3D and is considered a mechanism by which to encode more functions into the limited coding capacity of the virus (14). Other independent functions of 3CD and its influence on host cells require further exploration.

Cellular extrinsic factors such as hypoxia, nutritional deprivation, viral infection, and cell-intrinsic stresses such as endoplasmic reticulum (ER) stress, will activate the complex signaling pathways in eukaryotic cells integrated stress response (ISR). The intersection of all ISR responses is the phosphorylation of the α-subunit of eukaryotic initiation factor 2 (eIF2α). The eIF2, GTP, and Met-tRNAi combine to form a ternary complex (TC), a key step in translation initiation. After identifying the start codon, GTP is hydrolyzed into GDP. Subsequently, eIF2-GDP is recycled to eIF2-GTP by the GEF of eIF2, namely, eIF2B. Phosphorylated eIF2α (p-eIF2α) interferes with eIF2-GDP/GTP exchange through a stronger interaction with eIF2B, leading to decreased TC levels and translational inhibition. However, some mRNAs that continue to be translated when eIF2α is phosphorylated, such as *ATF4*, *GADD34*, and *CHOP*, containing an upstream open reading frame (uORF) in the 5′-UTR of these resistance genes (15). The uORF is the translational regulatory element of the 5′-UTR region, including an uAUG and stop codon. By default, uORF is recognized by the 40S ribosomal subunit and suppresses the translation of the downstream main open reading frame (mORF). In the case of eIF2α phosphorylation caused by stress, the reduced TC concentration allows the 40S ribosomal subunit to bypass uORF-AUG, thereby lifting the inhibition of mORF, so protein translation increases. The uORFs are ubiquitous in mammalian transcripts, but the uORFs complex regulatory mechanisms require more in-depth research.

Growth arrest and DNA damage-inducible protein 34 (GADD34, also known as PPP1R15A, MyDll6), is upregulated as a result of various cell stress inducing stimuli (16). GADD34, as the regulatory subunit of serine/threonine protein phosphatase 1 (PP1), can dephosphorylate eIF2α, restart protein synthesis, and help cells recover from ISR stress (17). GADD34 has complex regulatory effects on apoptosis, autophagy, and the mammalian target of rapamycin (mTOR) pathway. GADD34 also interacts with various cellular proteins to perform its functions (17, 18). In addition, GADD34 plays an important role in regulating the interferon response of virus-induced innate immunity (19, 20). It has been reported that GADD34 inhibits virus replication (19–23), oppositely GADD34 promotes the replication of infectious bronchitis virus (IBV) (24). Interestingly, several viruses encode viral proteins with regions of homology to GADD34, which can mimic the functions of GADD34 (25). At present, there is no report that GADD34 is involved in the replication of picornaviruses.

Enterovirus is able to take advantage of various host proteins to enhance its replication. Enterovirus hijacks host proteins by upregulating the expression of beneficial genes or recruiting supportive proteins to the ROs (26, 27). At present, through high-throughput screening methods, identifying key host factors that interact with the virus during the infection is essential for identifying genes that are selectively synthesized in the context of host shutoff and facilitating specific mechanistic research (3).

In this study, we investigated the interaction of EV71 replication with the host protein GADD34. After EV71 infects cells, the mRNA and protein levels of GADD34 are both upregulated. The activation of GADD34 could be detected after transfection of EV71 precursor protein 3CD, and the 3CD mutant Y441S or I631D did not increase GADD34 expression. The Y441S mutation within 3CD is lethal to EV71 infectious clones. 3CD upregulated GADD34 at RNA and protein levels. We detected that 3CD regulated the uORF2 of *GADD34* 5′-UTR in an eIF2α phosphorylation-independent manner to enhance the translation of *GADD34* mRNA.

GADD34 is necessary for EV71 replication as determined by overexpression and knockdown studies. The increase of viral replication stimulated by GADD34 is not related to the already established function of dephosphorylation of eIF2α. The viral replication stimulation requires the PEST 1, 2, and 3 regions of GADD34. The PEST repeats of GADD34 promotes the IRES activity of the EV71 5′-UTR and several other viruses. Finally, we report that the GADD34 563–565 aa interacts with the EV71 protein 3CD. This interaction assisted with the 3CD recruitment of GADD34 to the EV71 IRES. The studies reported here reveal new functions of the GADD34 and the viral protein 3CD. These results provide a new aspect of host-cell interactions.

## RESULTS

### The expression of GADD34 is upregulated during EV71 infection.

**FIG 1:**
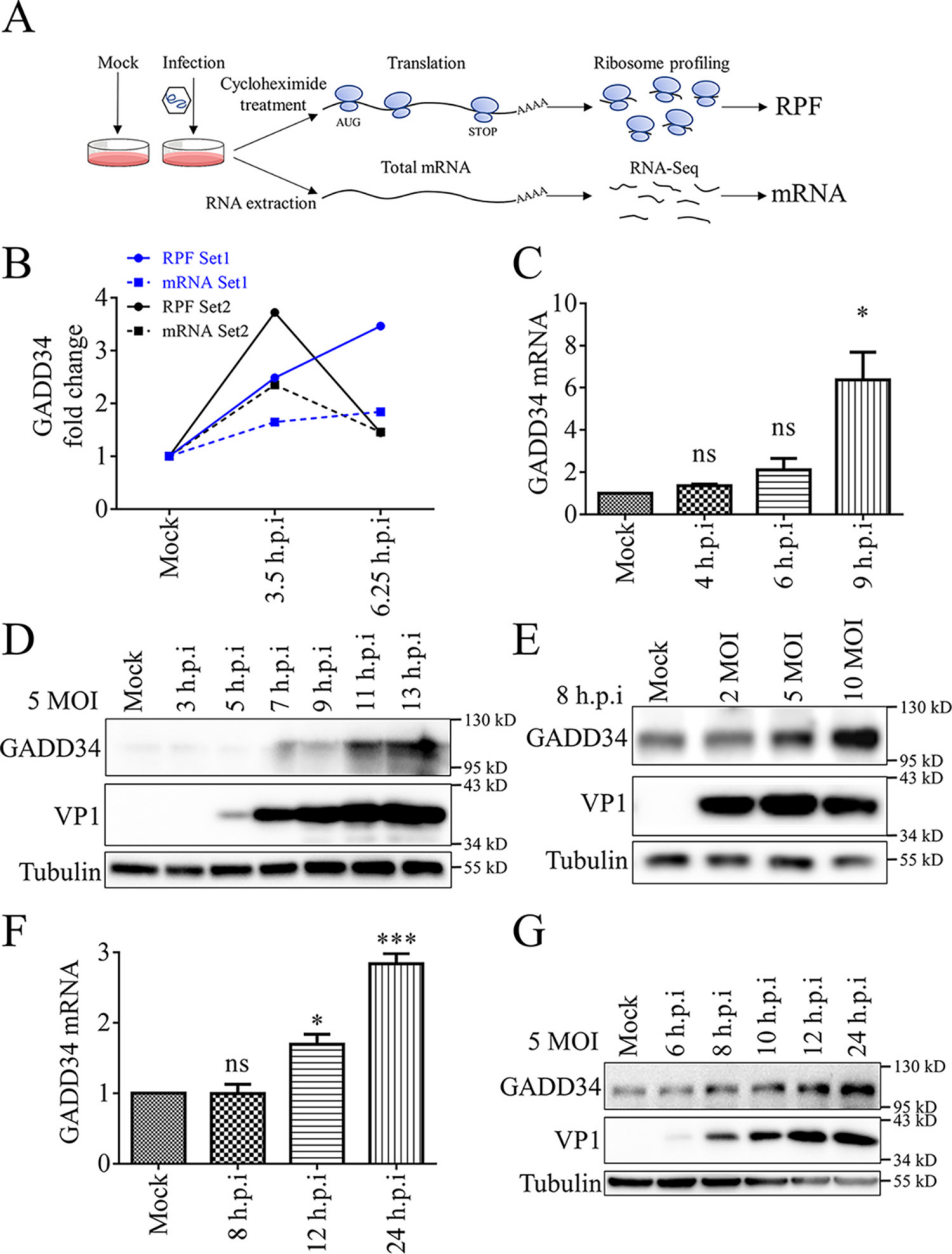
GADD34 identified by Riboseq screen to be upregulated during EV71 infection. (A) Riboseq and RNA-Seq screening processes. RD cells were infected with 40 MOI EV71 for 3.5 h and 6.25 h, compared with mock cells, to establish ribosome-protected fragment (RPF) and total mRNA libraries. (B) High-throughput screening results. The RPF and mRNA changes of GADD34 in the two biological replicates. (C) RD cells were infected with 10 MOI EV71, and the cells were harvested at different times after infection. GADD34 mRNA was detected by RT-qPCR. (D) After RD cells were infected with 5 MOI EV71 for a gradient time, GADD34 was detected by Western blotting. (E) RD cells were infected with EV71 with a gradient of MOI for 8 h, and the expression of GADD34 protein was detected by Western blotting. (F) HT-29 cells were infected with 5 MOI EV71, and the cells were harvested at different times after infection. GADD34 mRNA was detected by RT-qPCR. (G) After HT-29 cells were infected with 5 MOI EV71 for a gradient time, GADD34 was detected by Western blotting. Values were the means plus standard errors of the means (SEM) (error bars) from two individual experiments. Values were statistically evaluated compared to the mock control using a one-way ANOVA. *, *P < *0.05; ***, *P < *0.001; ns, not significant.

Previously, we used Riboseq (Ribosome Profiling), a high-throughput sequencing method, to analyze the changes in transcription and translation levels in rhabdomyosarcoma (RD) cells during EV71 infection (GSE103308) (28). Two libraries of the ribosome-protected fragment (RPF) and total cellular mRNA were constructed separately (Fig. 1A). RPF represents translated mRNA and RPF/mRNA is used to indicate translation efficiency (TE) of genes (15). At 3.5 h and 6.25 h after EV71 infection of RD cells, there were 21 genes that were differentially upregulated in both mRNA and RPF (Table S1 in the supplemental material). Among them, there were nine genes whose translation efficiency was upregulated (Table S2). GADD34 is a stress-related host gene whose RPF and mRNA levels were both upregulated after EV71 infection in the screening results (Fig. 1B). Screening results were first verified. RD cells were infected with EV71, and the mRNA and protein levels of GADD34 were stimulated as the infection progressed (Fig. 1C to E). Immunofluorescence analysis showed the upregulation of GADD34 (green fluorescence) was detected in RD cells infected with EV71, while the endogenous GADD34 of non-infected cells was too little to be detected (Fig. S1). In human intestinal HT-29 cells (Fig. 1F and G), Caco-2 cells (Fig. S2B) and human neuroblastoma SH-SY5Y cells (Fig. S2A), GADD34 was also induced by EV71 infection. These results indicate that the expression of cellular GADD34 was significantly upregulated during EV71 infection.

### GADD34 is upregulated by EV71 non-structural protein precursor 3CD.

**FIG 2:**
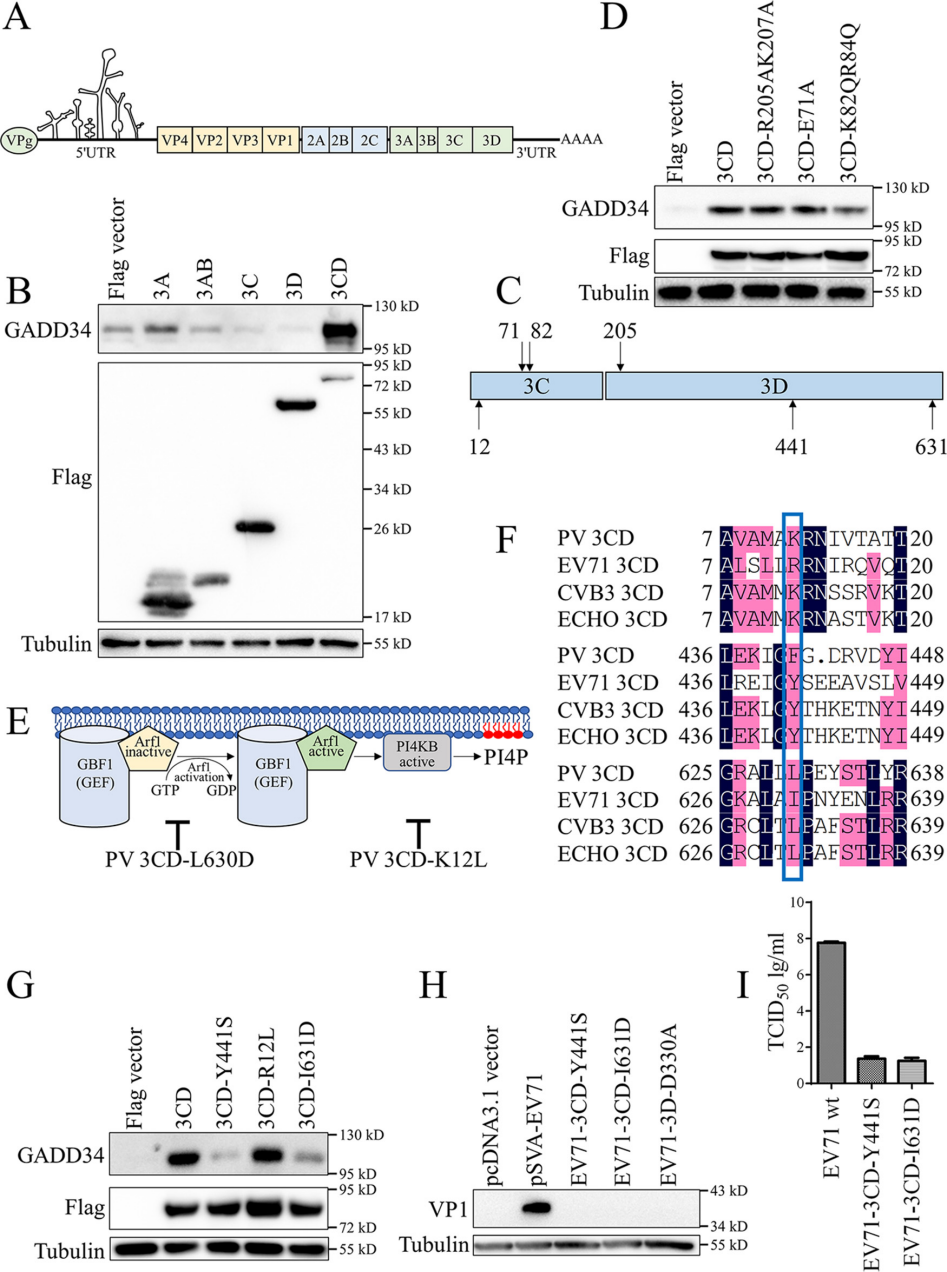
EV71 non-structural precursor protein 3CD upregulates GADD34. (A) Schematic diagram of EV71 genome structure. (B) Screen of the viral components that activate GADD34 protein. pCE-puro-3×Flag-3A, 3AB, 3C, 3D, and 3CD were transfected into HeLa cells, and GADD34 protein expression was detected by Western blotting after 48 h. (C) Schematic diagram of constructing 3CD mutation sites. (D) HeLa cells were transfected with EV71 3CD wild-type and 3CD nuclear localization signal mutation R205AK207A, protease activity mutation E71A, and RNA binding mutation K82QR84Q, GADD34 was detected by Western blotting 48 h after transfection. (E) The mutants L630D and K12L of poliovirus 3CD interfere with different stages of Arf-PI4P activation. (F) R12, Y441, and I631 of EV71 3CD are conserved in enterovirus. (G) HeLa cells were transfected with EV71 3CD wild-type and 3CD mutant Y441S, R12L, or I631D, GADD34 was detected by Western blotting 48 h after transfection. (H) Plasmids infectious clone pSVA-EV71, pSVA-EV71-3CD-Y441S, pSVA-EV71-3CD-I631D, or pSVA-EV71-3D-D330A were cotransfected into HeLa cells, respectively, with T7 RNA polymerase (plasmid pBABE). Cells were harvested 48 h after transfection, and Western blotting was used to detect VP1 to characterize the protein production of EV71. (I) The titers of virus EV71 wild-type and 3CD mutant virus were measured by TCID_50_. Values were the means plus standard errors of the means (SEM) (error bars) from two individual experiments.

Initial experiments were designed to identify which components of EV71 induced GADD34 expression. As shown in Fig. 2A, EV71 encodes one polyprotein precursor, which eventually forms 11 mature proteins. Expression plasmids of four structural proteins and seven non-structural proteins were transfected into HeLa cells. The non-structural protein 3A slightly induce GADD34 protein expression (Fig. S3). We hypothesized that P3 (the precursor proteins of 3A, 3B, 3C, and 3D) was an important factor for activating GADD34. Flag-tagged 3A, 3AB, 3C, 3D, and 3CD were transfected into HeLa cells, overexpression of the precursor protein 3CD induced GADD34 protein expression (Fig. 2B). The immunofluorescence results confirmed that the green fluorescence of GADD34 could be detected only in the HeLa cells overexpressing Flag-3CD (Fig. S4). Functional sites on 3CD were identified and mutagenesis clones at these sites were constructed (Fig. 2C). 3CD exhibited different specific protease activity and RNA binding activity from 3C (9). The glutamic acid at position 71 of the active protease center was mutated to alanine, and the RNA binding domain ^82^KFRDI^86^ was mutated to ^82^QFQDI^86^ (29, 30). In addition, a potential NLS domain ^203^PTRTKLEPS^211^, which was highly similar to the human rhinovirus (HRV), a member of the *Picornaviridae* family, was mutated to ^203^PTATALEPS^211^ (31). The above three mutants were transfected into HeLa cells, and compared with wild-type 3CD, the mutants still activated the expression of GADD34 (Fig. 2D).

Because protease activity, RNA binding activity, and nuclear localization are not contributed to the upregulation of GADD34 by 3CD, we sought to find clues from other enteroviruses. Studies had shown that the 3CD of poliovirus (PV) could induce Arf to bind to the membrane resulting in an activated GTP-bound Arf form. The F441S mutation in PV 3CD led to the loss of Arf-activating function (12, 13). Another report indicated that the expression of PV 3CD was sufficient to induce the phosphatidylinositol-4-phosphate (PI4P), a marker of RNA virus ROs. PI4P induction required GBF1, Arf1-GTP, and activated Phosphatidylinositol 4-kinase beta (PI4KB). The mutation in the 3D domain of PV 3CD, L630D, could interfere with the steps before Arf1 was activated, while the mutation in the 3C domain, K12L, could interfere with the steps after Arf1 was activated (2) (Fig. 2E). We compared the amino acid sequence of PV 3CD with the 3CD of other members of the *enterovirus* genus, including EV71, coxsackievirus B3 (CVB3), and echovirus (ECHO). The amino acids F441, K12, and L630 of PV 3CD were relatively conserved in *enterovirus* (Fig. 2F). Based on this, we constructed three single-point mutants, EV71 3CD Y441S, R12L, and I631D. The results of HeLa cells transfection showed that EV71 3CD mutants Y441S and I631D could significantly impair the activation of GADD34 by 3CD, but there was no significant difference between R12L and wild-type (Fig. 2G). To explore whether the upregulation of GADD34 by EV71 3CD depends on Arf1, HeLa cells were treated with Brefeldin A (BFA), an inhibitor of Afr1. The results showed that GADD34 was still activated by 3CD with BFA (Fig. S5), indicated that the Arf1 was not essential for the upregulation of GADD34 by 3CD.

We then examined whether the functional site of EV71 3CD upregulating GADD34 could correspond to the live virus. EV71 infectious clones were constructed with the 3CD-Y441S and I631D mutant, and the RdRp activity loss mutant 3D-D330A was used as a control (32). The infectious clone and mutant plasmids were transfected into the cells, and the results of Western blotting and viral titration indicated that 3CD-Y441S and I631D would lose the replication of EV71 (Fig. 2H and I). We could not determine the contribution of 3CD-Y441S or I631D to the upregulation of GADD34 during EV71 infection due to its lethality, but it was certain that these sites were necessary for virus replication. In short, overexpression of EV71 3CD, only one precursor protein, could upregulate the expression of GADD34. The two EV71 3CD mutants Y441S and I631D that lost the activation function of Arf also weakened the activation function of GADD34.

### GADD34 is upregulated by EV71 3CD at both mRNA and protein levels.

**FIG 3:**
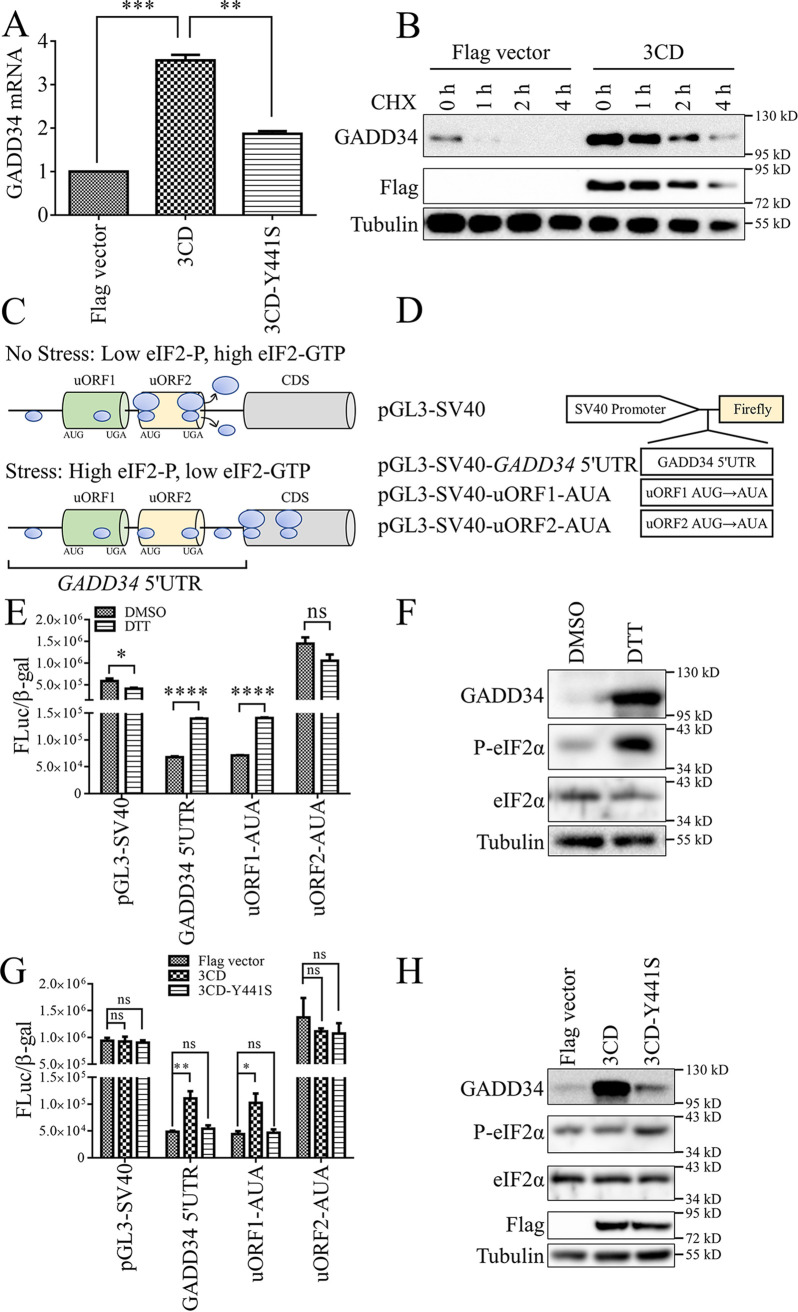
EV71 3CD upregulates the mRNA and protein of GADD34. (A) HeLa cells were transfected with pCE-puro-3×Flag-3CD and 3CD-Y441S. After 48 h, cells were harvested, and the mRNA level of *GADD34* was detected by RT-qPCR. (B) The EV71 3CD was transfected into HeLa cells, and the cells were harvested 30 h after transfection to detect the level of GADD34 protein by Western blotting. Before being harvested, cells were treated with 10 μg/mL cycloheximide (CHX) for 0 h, 1 h, 2 h, and 4 h. (C) In the presence or absence of stress, the effect of uORF2 in the 5′-UTR region on the translation of the downstream main open reading frame (mORF). (D) *GADD34* 5′-UTR was constructed into a pGL3-SV40 promoter vector. On this basis, the AUG of two uORFs in the *GADD34* 5′-UTR region was mutated to AUA, respectively. (E and F) The plasmids constructed in (D) were transfected into HeLa cells, and the cells were harvested 30 h later. Before cell harvest, cells were treated with dimethyl sulfoxide (DMSO) or 10 mM dithiothreitol (DTT) for 6 h. Cell lysates were used to detect FL and β-galactosidase (β-gal) activities (E) and to detect proteins by Western blotting (F). (G and H) The plasmids constructed in (D) and EV71 3CD or 3CD-Y441S were co-transfected into HeLa cells. 48 h after transfection, cell lysates were used to detect FL and β-gal activities (G) and to detect protein by Western blotting (H). Values were the means plus standard errors of the means (SEM) (error bars) from two (A) or three (E and G) individual experiments. Values were statistically evaluated using a one-way ANOVA (A and G) or a two-tailed *unpaired t test* (E). *, *P < *0.05; **, *P < *0.01; ***, *P < *0.001; ****, *P < *0.0001; ns, not significant.

Further studies were conducted to determine at what level EV71 3CD activated GADD34. When 3CD was overexpressed in HeLa cells, an increase in GADD34 mRNA is detected, and 3CD-Y441S could inhibit this upregulation (Fig. 3A). The results indicated that 3CD increased the mRNA level of GADD34.

Next, the effect of 3CD on the protein level of GADD34 was tested. 3CD was confirmed to not affect the stability of the GADD34 protein by adding cycloheximide (CHX), a drug that hinders protein translation, to the cell culture medium (Fig. 3B). We speculated that 3CD upregulated the translation of GADD34. The regulation of gene expression, including translation regulation, are essential for adaptation and survival under stress conditions. The 5′-UTR of mRNA regulates the translation initiation of stress-induced proteins. The IRES and uORF represent functional elements present within the 5′-UTR.

The dual-luciferase reporter plasmid is often used as the gold standard for detecting whether 5′-UTR has IRES activity (33). For the pRHF vector, the upstream *Renilla* luciferase (RL) and the downstream firefly luciferase (FL) are on the same transcribed mRNA (see Fig. S6A in the supplemental material). The translation initiation of RL is cap-dependent, and translation will end at the stop codon and hairpin structure, resulting in low translation levels of downstream FL. If the 5′-UTR fragment inserted between the two luciferases has the IRES activity to recruit ribosomes, the downstream FL activity will increase. FL/RL is a standard tool used to show IRES activity. We inserted the 5′-UTR of *GADD34* into the pRHF vector, and dual-luciferase analysis showed that *GADD34* 5′-UTR had IRES activity (Fig. S6B). The results of 3CD transfection showed that 3CD would not affect the IRES activity of *GADD34* 5′-UTR obviously (Fig. S6C).

A previous report stated that the human *GADD34* 5′-UTR contains two non-overlapping uORFs (34). In the absence of stress stimulation, the ribosome scans the 5′-UTR of *GADD34* mRNA and initiates translation at uORF2, resulting in a low translation level of the main open reading frame CDS. When stress occurs, the ribosome scans across the uORF and initiates translation at the CDS, which results in massive translation of the GADD34 protein (35) (Fig. 3C). We inserted *GADD34* 5′-UTR into a luciferase reporter plasmid with SV40 promoter and mutated the AUG of the two uORFs into AUA, respectively (Fig. 3D). Consistent with previous studies, dithiothreitol (DTT) induced the phosphorylation of eIF2α and the upregulated expression of GADD34, and at the same time, the activity of *GADD34* 5′-UTR was activated (34, 35) (Fig. 3E and F). In addition, the uORF1 mutant had similar activity to the wild type and was also induced by DTT, but the basic activity of the uORF2 mutant was approximately 20 times higher than that of the wild type, and it was no longer activated by DTT (Fig. 3E). These results indicated that uORF2 played a dominant role in GADD34 translation regulation, including inhibiting basic translation when there was no stress and inducing translation in response to stress (34). We examined the effect of EV71 3CD on *GADD34* uORF translation regulation. The results showed that 3CD could activate the 5′-UTR of *GADD34*, and similar to DTT, helped to bypass the inhibitory uORF2, and this effect would be offset by the Y441S mutant (Fig. 3G). Different from DTT induction, 3CD did not cause enhanced phosphorylation of eIF2α (Fig. 3H).

In summary, the above results indicate that EV71 3CD upregulates the expression of GADD34 at both mRNA and protein levels, suggesting that 3CD has a coupled function to regulate at both mRNA and protein levels.

### GADD34 promotes EV71 replication.

**FIG 4:**
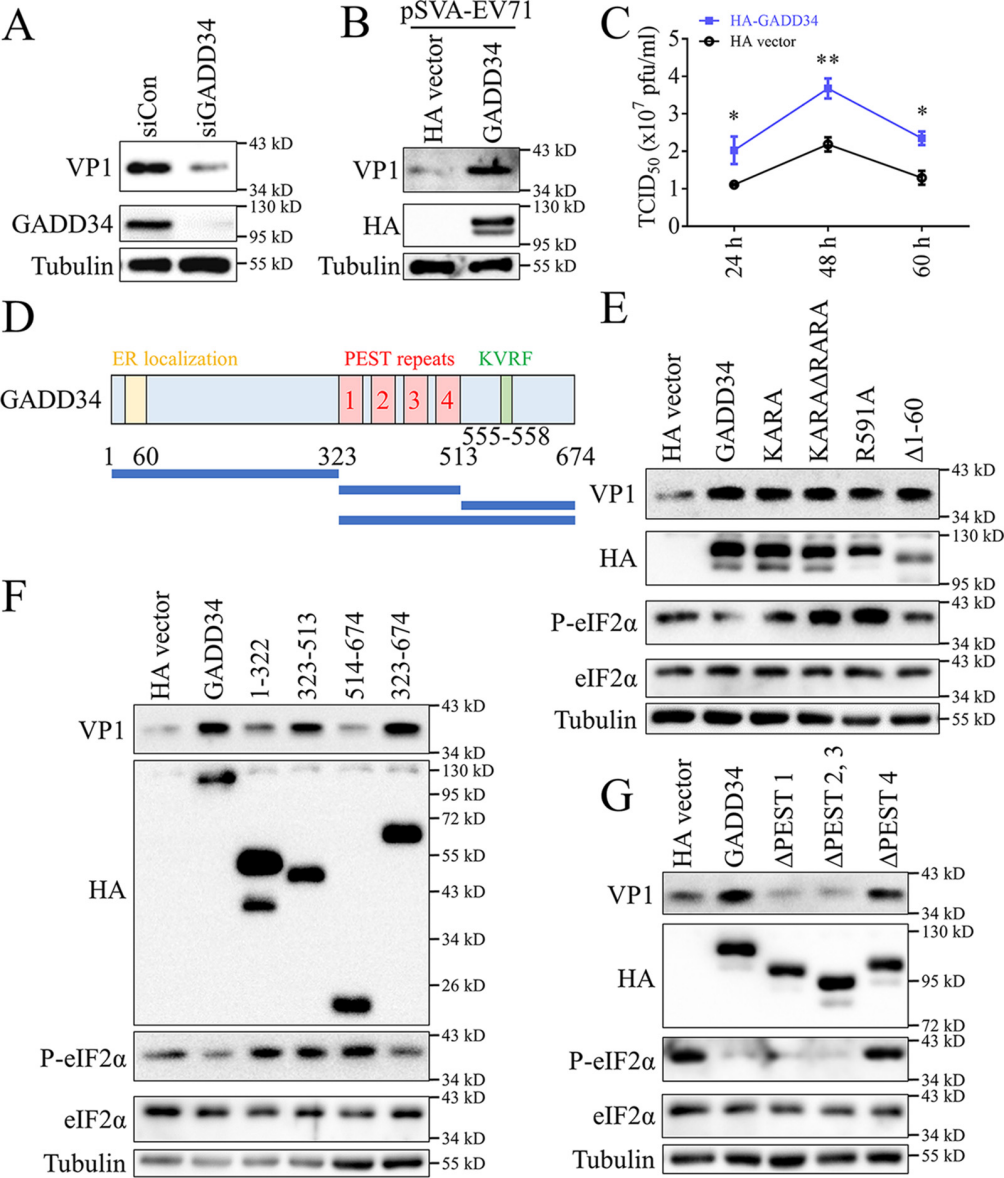
GADD34 promotes EV71 replication, which was related to its PEST repeats area. (A) RD cells were transfected with siCon or siGADD34. 24 h after transfection, cells were infected with 1 MOI EV71 for 8 h. In harvested cells, proteins were detected by Western blotting. (B) The infectious clone pSVA-EV71, T7 RNA polymerase, and pCMV-3×HA vector or pCMV-3×HA-GADD34 were cotransfected into RD cells. Cells were harvested 48 h later, and the expression of EV71 VP1 was detected by Western blotting. (C) The HA vector or HA-GADD34 was transfected into RD cells. Cells were infected with 0.1 MOI EV71 24 h after transfection, and the culture supernatant was collected at 24 h, 48 h, and 60 h postinfection. The viral titers were measured by endpoint titration analysis and expressed as 50% tissue culture infectious dose (TCID_50_). Values were the means plus standard errors of the means (SEM) (error bars) from three individual experiments. Values were statistically evaluated using a two-way ANOVA. *, *P < *0.05; **, *P < *0.01. (D) Schematic diagram of GADD34 protein structure. The truncations are shown. (E to G) HA-GADD34 wild-type or various mutants, deletions, and truncations were transfected into RD cells. Cells were infected with 1 MOI EV71 24 h after transfection. Then cells were harvested 8 h after infection to detect proteins by Western blotting.

The previous results showed the mechanism by which EV71 infection upregulated GADD34. Furthermore, we tried to explore whether GADD34 affected EV71 replication. In Jurkat and RD cells, endogenous GADD34 was knocked down, and the cells were infected with EV71. Detection of EV71 viral RNA (vRNA) by RT-qPCR showed that after knocking down GADD34, EV71 RNA was also reduced (Fig. S7). The same results were obtained in RD cells by detecting the virus structural protein VP1 by Western blotting (Fig. 4A). These results indicated that GADD34 is required for efficient EV71 replication. The EV71 infectious clone plasmid pSVA-EV71 and GADD34 expression plasmid (HA tag) were transfected into RD cells, and the amount of VP1 in the cell lysate was detected. The results showed that VP1 was increased in cells overexpressing GADD34 (Fig. 4B). This result indicated that GADD34 promoted the EV71 replication process inside the cells rather than entry or release. The titers of viruses were measured using a 50% tissue culture infective dose (TCID_50_) assay. The results showed that EV71 titer was higher in the supernatant of RD cells overexpressing GADD34 (Fig. 4C). In summary, GADD34 promotes EV71 replication.

Studies were conducted to identify the function of GADD34 promoted EV71 replication. As a scaffold, GADD34 recruits the PP1 and eIF2α, then dephosphorylates eIF2α, thereby restoring normal cellular protein synthesis after stress (36). Fig. 4D is a schematic diagram of the main domains of the GADD34 protein. GADD34 interacts with PP1 through ^555^KVRF^558^ and ^612^RARA^615^ motifs (37) and interacts with eIF2α through the ^591^RFARR^595^ motif (38). In addition, the N-terminal of GADD34 mediates its ER localization (39). The GADD34 mutants KARA (^555^KVRF^558^→^555^KARA^558^) and KARAΔRARA (^555^KVRF^558^→^555^KARA^558^ and delete ^612^RARA^615^) that were defective in the interaction with PP1, the mutant R591A that was defective in interaction with eIF2α, and the mutant Δ1-60 with deletion of ER localization, were constructed and transfected into RD cells. The results of EV71 infection showed that all mutants could still promote virus replication, although KARA, KARAΔRARA, and R591A no longer had the same dephosphorylation function of eIF2α as the wild type (Fig. 4E), indicating that the promotion of EV71 replication by GADD34 was not related to the phosphorylation status of eIF2α, nor the ER localization of GADD34.

A series of GADD34 truncations were constructed to identify functional domains that promoted EV71 replication (Fig. 4D). Western blotting results showed that truncations 323 to 513 and 323 to 674 still promoted EV71 replication (Fig. 4F). In addition, GADD34 ΔPEST no longer promotes EV71 replication (Fig. S8). It was indicated that the PEST repeat region domain 323 to 513 was sufficient to promote EV71 replication. To study the function of the four PEST repeats, we constructed three deletion mutants, ΔPEST 1, ΔPEST 2, 3, and ΔPEST 4, respectively. The results of EV71 infection displayed that ΔPEST 1 and ΔPEST 2, 3 would destroy the stimulation effect of GADD34 on EV71, while ΔPEST 4 did not affect (Fig. 4G). Based on the above results, GADD34 promoted the replication of EV71, and its main functional area was the PEST repeats, of which PEST 1, 2, and 3 contributed the most.

### GADD34 promotes the IRES activity of EV71 5′-UTR.

**FIG 5:**
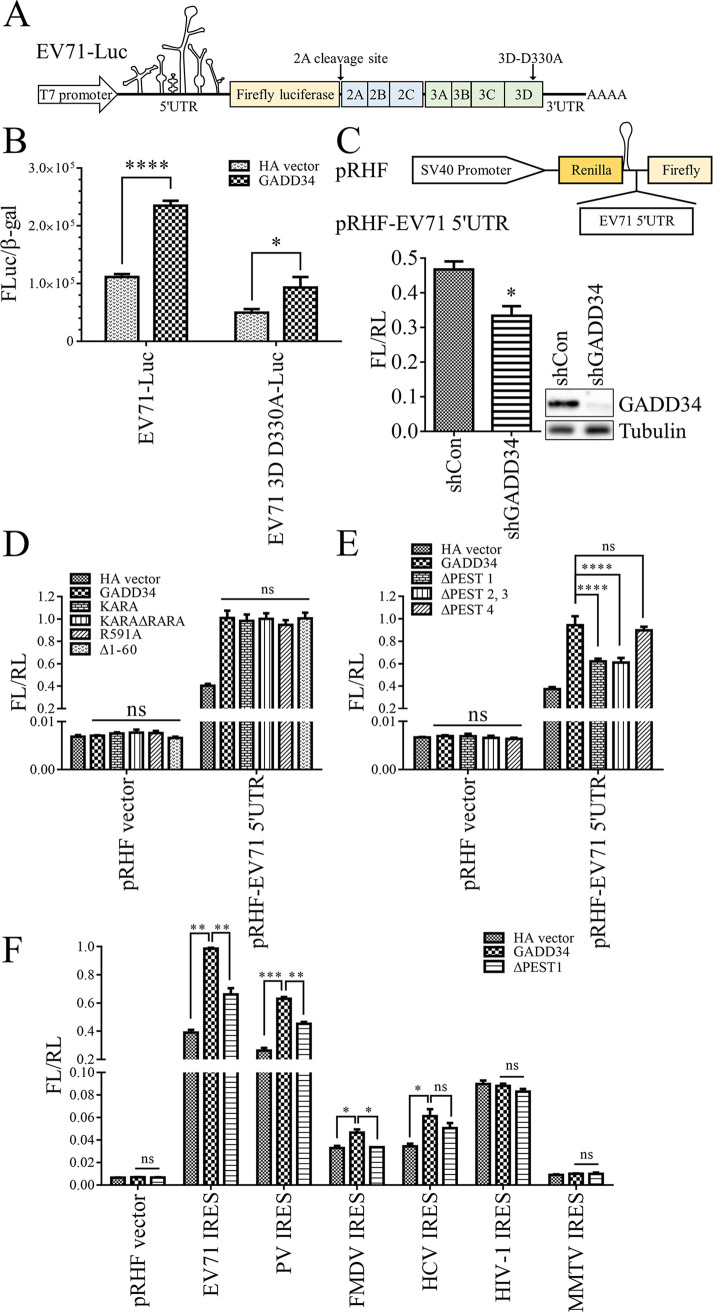
GADD34 promotes EV71 IRES-mediated translation. (A) Schematic diagram of EV71 subgenomic replicon EV71-Luc. (B) The plasmids EV71-Luc or EV71-3D-D330A-Luc and HA vector or HA-GADD34 were cotransfected into HEK293T cells, then FL and β-gal activities were detected 48 h later. (C) HeLa cells were infected with pSIREN-RetroQ-shGADD34 or shControl (shCon) packaged lentivirus. Two days later, cells were transfected with pRHF-EV71 5′-UTR. Cells were collected 48 h after transfection to detect FL and RL activities, and Western blotting was used to detect the knockdown effect of GADD34. (D and E) HA-GADD34 wild-type or various mutants and pRHF-EV71 5′-UTR were transfected into HeLa cells. FL and RL activities were detected after 48 h. (F) HA-GADD34 wild-type or HA-GADD34 ΔPEST 1 and dual-luciferase plasmids containing several viral IRESs were cotransfected into HeLa cells. FL and RL activities were detected after 48 h. Values were the means plus standard errors of the means (SEM) (error bars) from two (F) or three (B, C, D, and E) individual experiments. Values were statistically evaluated using a two-way ANOVA (B, D, and E) or a two-tailed *unpaired t test* (C) or a one-way ANOVA (F). *, *P < *0.05; **, *P < *0.01; ***, *P < *0.001; ****, *P < *0.0001; ns, not significant.

The IRES-mediated translation and viral genome RNA replication are two key stages in the intracellular life cycle of EV71. To explore the effect of GADD34 on the translation or RNA replication of EV71, we transfected the EV71 subgenomic replicon (a gift plasmid from Professor Wenhui Li [40]) and T7 RNA polymerase into HEK293T cells. In the EV71 subgenomic replicon EV71-Luc, the structural protein precursor P1 (the precursor of VP1-4) was replaced by luciferase (Fig. 5A). This replicon can translate and replicate the genome (41) but cannot form complete progeny virus particles. We mutated aspartic acid at position 330 of the 3D RdRp active site of EV71-Luc to alanine (Fig. 5A). The EV71-Luc mutant D330A can only perform IRES-dependent translation and can no longer replicate RNA (42). The luciferase activity of wild-type and mutant D330A replicons could be upregulated by GADD34 (Fig. 5B), indicating that GADD34 could activate EV71 IRES-mediated translation. To further confirm the finding, we overexpressed the dual-luciferase reporter plasmid pRHF-EV71 5′-UTR in HeLa cells. In the plasmid pRHF-EV71 5′-UTR, RL is the cap-dependent translation, and FL is the EV71 IRES-dependent translation (for more details, please refer to Fig. S6A). When GADD34 was knocked down, the reporter plasmid FL/RL decreased (Fig. 5C), indicating that GADD34 could activate the activity of EV71 IRES.

To find the functional domain where GADD34 activated EV71 IRES, mutants that lacked the interaction with PP1 and eIF2α or lacked the ER localization signal were studied. Cotransfection of the mutants and pRHF-EV71 5′-UTR plasmids showed that all mutants had the same ability to activate EV71 IRES as the wild type (Fig. 5D). The function of the PEST repeats was also tested. ΔPEST 4 had the same effect as wild-type in activating EV71 IRES, but the activation effect of ΔPEST 1 or ΔPEST 2, 3 were significantly weakened (Fig. 5E). These results indicated that similar to the results of EV71 infection (Fig. 4E to G), GADD34 activated EV71 IRES, and its PEST 1, 2, and 3 domains were required but were not related to its function of eIF2α dephosphorylation.

According to its requirements for eIF, viral IRESs can be divided into four main classes (43). Type I poliovirus (PV) IRES (44), type II foot-and-mouth disease virus (FMDV) IRES (45), type III Hepatitis C virus (HCV) IRES (46), and unclassified human immunodeficiency virus type 1 (HIV-1) IRES (47) and mouse mammary tumor virus (MMTV) IRES (48) were selected to verify the functionality of GADD34. GADD34 or its mutant ΔPEST 1 and each viral IRES were cotransfected in HeLa cells. The results showed that GADD34 activated IRES of EV71, PV, FMDV, HCV, and ΔPEST 1 weakened the activation (Fig. 5F). In summary, GADD34 PEST repeats contributed to the activation of IRES of several viruses.

### GADD34 interacts with EV71 non-structural protein precursor 3CD.

**FIG 6:**
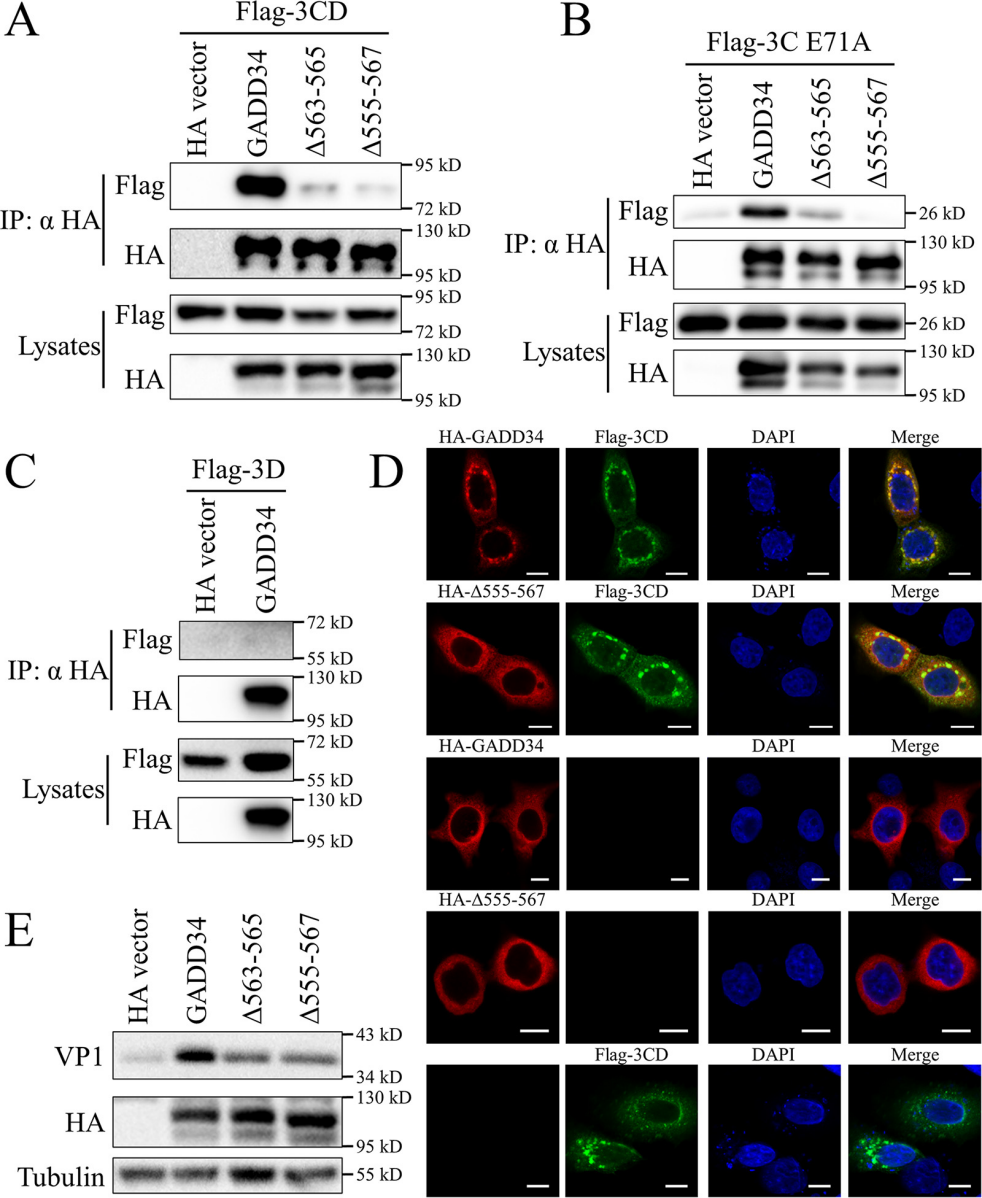
The C-terminal of GADD34 interacts with EV71 3CD. (A to C) 3×Flag-3CD (A), 3×Flag-3C-E71A (B), or 3×Flag-3D (C) and HA-GADD34, HA-GADD34-Δ563-565, or HA-GADD34-Δ555-567 were co-transfected into HEK293T cells. After 48 h, cells were lysed, the proteins were collected by co-immunoprecipitation and detected by Western blotting. (D) HA-GADD34 or HA-GADD34-Δ555-567 and 3×Flag-3CD were co-transfected into HeLa cells. After 48 h, cells were fixed, and the protein localization was detected by immunofluorescence. Bars represent 10 μm. (E) HA vector, HA-GADD34, HA-GADD34-Δ563-565, or HA-GADD34-Δ555-567 was transfected into RD cells. After 24 h, cells were infected with 1 MOI EV71 for 8 h, and the viral protein VP1 was detected by Western blotting.

In Fig. 2 and 3, the mechanism of EV71 3CD upregulating GADD34 is studied. To explore whether 3CD and GADD34 has a deeper functional connection, we tested whether there was an interaction between the two. We cotransfected GADD34 or the truncations and EV71 3CD in HEK293T cells for co-immunoprecipitation. After a series of GADD34 truncation verification, we located the interaction area in GADD34 555–567 aa (data not shown). The co-immunoprecipitation results indicate that GADD34 interacts with EV71 3CD, and the interaction was realized by 555 to 567 aa of GADD34 (Fig. 6A). In the 3CD structure, the 3C and 3D domains are separated by a short linker (180 to 186 aa), and there is no interaction between the two domains (14). Through co-immunoprecipitation, evidence of GADD34 interaction with 3C instead of 3D was demonstrated (Fig. 6B and C). Immunofluorescence analysis in HeLa cells confirmed the interaction of GADD34 and EV71 3CD as co-localization of the two was observed, and the mutation Δ555-567 disrupted the co-localization (Fig. 6D). The effect of the interaction of GADD34 and 3CD on the replication of EV71 was studied. Western blotting results showed that the mutants Δ563-565 and Δ555-567 lacking 3CD interaction maintained EV71 replication stimulation, but compared with the wild type, the replication of EV71 was significantly weakened (Fig. 6E). The above results indicated that GADD34 interacted with EV71 3CD, and this interaction contributed to GADD34's promotion of EV71 replication.

### EV71 3CD recruits GADD34 to EV71 5′-UTR.

**FIG 7:**
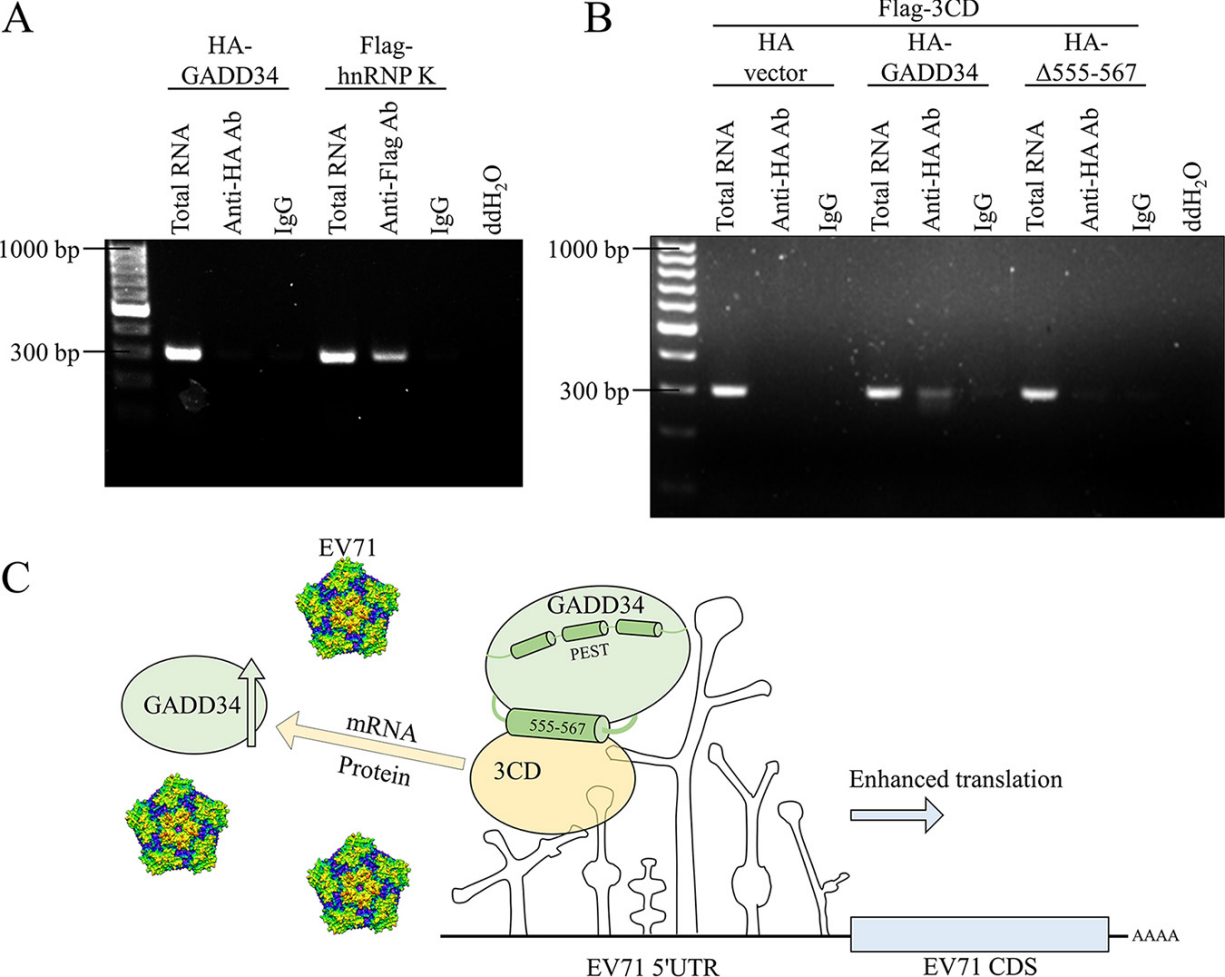
EV71 3CD recruits GADD34 to EV71 5′-UTR. (A) HA-GADD34 or 3×Flag-hnRNP K and pRHF-EV71 5′-UTR were cotransfected into HEK293T cells. After 48 h, cells were lysed for RNA-protein immunoprecipitation (RIP). Agarose gel electrophoresis showed the amount of cDNA reverse-transcribed from the precipitated RNA. (B) HA vector, HA-GADD34, or HA-GADD34-Δ555-567 and pRHF-EV71 5′-UTR and 3×Flag-3CD were cotransfected into HEK293T cells. After 48 h, cells were lysed for RIP. Proteins were precipitated by anti-HA antibody and beads, and the cDNA reverse-transcribed from the pulled RNA was detected by agarose gel electrophoresis. (C) Summary chart of this study. EV71 upregulated the RNA and protein of host protein GADD34 through the non-structural precursor 3CD. 3CD recruited GADD34 to EV71 5′-UTR by interacting with GADD34 to promote IRES-mediated viral protein translation. GADD34 555 to 567 aa mediated the interaction with EV71 3CD, and PEST repeats contributed to the enhancement of IRES activity.

The 3CD of the picornavirus binds to the cloverleaf structure of the viral RNA 5′-UTR (49). We hypothesized that 3CD contributed to the recruitment of GADD34 to the 5′-UTR during EV71 infection, thereby helping GADD34 promote IRES-mediated translation initiation. RNA immunoprecipitation (RIP) results showed that GADD34 could not directly bind to EV71 5′-UTR, and the heterogeneous nuclear ribonucleoprotein K (hnRNP K), a protein known to bind to EV71 5′-UTR (50), was used as a positive control (Fig. 7A). Then, three plasmids, GADD34, 3CD, and pRHF-EV71 5′-UTR, were cotransfected into HEK293T cells. The RIP results showed that GADD34 could precipitate EV71 5′-UTR through the intermediate linker 3CD, while GADD34 mutant Δ555-567 was attenuated (Fig. 7B). The precipitated EV71 5′-UTR was quantified by RT-qPCR, and the same results were obtained (Fig. S9). These results indicated that EV71 3CD assisted with the recruitment of GADD34 to the EV71 5′-UTR region.

The summary of this study was shown in Fig. 7C. When EV71 infected cells, on the one hand, EV71 non-structural precursor protein 3CD upregulated the expression of GADD34 at both mRNA and protein levels; on the other hand, 3CD recruited GADD34 to the vicinity of the EV71 5′-UTR, and the PEST repeats domain of GADD34 could enhance the IRES activity of the EV71 5′-UTR, thereby promoting EV71 replication.

## DISCUSSION

At present, many studies have analyzed the differential expression of host genes after EV71 infection through high-throughput screening, which is conducive to screening out novel host proteins that perform outstanding functions during infection (51–57). These studies mainly applied both transcriptome and proteomics screening techniques. Our study is the first to use ribosome profiling and RNA-sequencing (RNA-Seq) to analyze and screen host genes whose mRNA and TE have changed significantly during EV71 infection. Picornavirus infection is characterized by shutting down host transcription and protein synthesis. When the overall translation of the cell is inhibited, we screen the host proteins with elevated translation levels, which will help in understanding the basic cell biology and how cell resources are used by viruses during replication.

The dominant factor for EV71 infection to activate the host protein GADD34 is mapped to the non-structural protein precursor 3CD in research. The overexpression of EV71 3CD in HeLa cells can lead to a large increase in the expression of GADD34, while the monomeric proteins 3C and 3D cannot, indicating that this is a unique effect of 3CD. We conducted studies that demonstrated EV71 3CD mutants Y441S and I631D remove the activation of GADD34, which seems to be similar to the activation of Afr-PI4P by PV 3CD (2, 12, 13), which prompted us to consider whether there is a common upstream mechanism for enterovirus 3CD to upregulate the expression of PI4P and GADD34. Enterovirus 3CD may enhance the expression of other proteins required for viral replication by activating Arfs or a similar mechanism. Especially membrane-bound proteins or proteins recruited to the vesicles of the replication complex. However, the results showed that GADD34 was still activated by 3CD with BFA, an inhibitor of Afr1, indicated that the Arf1 is not essential for the upregulation of GADD34 by 3CD (Fig. S5). Combined with no reports on GADD34 involved in Arf1 pathway, we think that the activation of Afr1 and GADD34 by 3CD are two parallel downstream events. These hypotheses require high-throughput screening experiments to explore and more examples of host proteins upregulated by 3CD to support it. This study has made significant findings which expand the understanding of the unique functions of enterovirus 3CD.

As a short-lived protein, GADD34 has a half-life of less than 1 h (16). GADD34 has a low endogenous background and is expressed in large quantities under stress. GADD34 expression is regulated by multiple steps: transcription, translation, and protein stability. The uORF is common in the 5′-UTR of genes resistant to eIF2 inhibition (15, 35). There have been several reports about the uORF of *GADD34* regulating its translation (34, 35). Gerresheim et al. used ribosome profiling to analyze that when HCV is infected, HCV can cause eIF2 inhibition like EV71, the intracellular *GADD34* mRNA is regulated by uORF-mediated translation (58). Similarly, our results use ribosome profiling to identify that EV71 infection upregulates the TE of GADD34. In addition, the results support that EV71 3CD regulates the uORF of *GADD34*; this regulation is independent of eIF2α phosphorylation, which is different from DTT-induced eIF2α phosphorylation to control uORF. We hypothesize that this may be due to the role that EV71 3CD regulates the GEF (eIF2B) of eIF2 in a similar mechanism to modulates the GEFs of Arfs, so that bypassing eIF2α phosphorylation, but directly affecting the GTP-GDP transformation to regulate uORF. In summary, our results expand our understanding of the regulation of *GADD34* uORF and provide an example of a viral protein regulating host protein translation. Moreover, 3AB and 3CD are the main cleaved products of P3 (the precursor proteins of 3A, 3B, 3C, and 3D), and the amount is more than the minor products 3C and 3D (2, 11), which provides evidence that there is sufficient 3CD in the infected cells to function.

Researchers usually focus on the function of host proteins activated by a viral infection in during viral replication. Shang et al. found that EV71 infection induces Sox4 expression, and Sox4 shuts down host immunity by inhibiting TLR signaling, promoting virus replication (26). Song et al. reported that the EV71 cascade activates EGR1 expression, and the activated EGR1 regulates the IRES of EV71 to enhance virus replication (27). In this study, GADD34, as a host protein upregulated by EV71 infection, was also found to promote EV71 replication. The function of GADD34 is routinely found as a regulatory subunit of PP1, which dephosphorylates eIF2α, restores translation and protein homeostasis (17, 36). GADD34 affects IBV, Chikungunya virus (CHIKV), Ebola virus (EBOV), and vesicular stomatitis virus (VSV) through eIF2α phosphatase activity (19, 20, 22, 24). However, the study by Ishaq et al. found that the inhibition of HIV-1 by GADD34 is not related to its well-known eIF2α phosphatase activity (23). Our results also provide significant evidence that GADD34 promotes EV71 replication independently of its eIF2α phosphatase function. Some previous studies have discussed that GADD34 may have other functions besides controlling eIF2α (17, 37). GADD34 binds various proteins, and its roles are not limited to regulating protein translation (17).

We report here for the first time that GADD34 can promote viral IRES-dependent translation initiation, which suggests that the function of GADD34 is not limited to restoring cap-dependent translation during stress but also contributes to promoting cap-independent translation. Ishaq et al. found that GADD34 inhibits HIV-1 5′-UTR RNA-mediated translation, but not by affecting HIV-1 IRES (23). Our research also concluded that GADD34 could not affect HIV-1 IRES, but GADD34 promotes classic I to III types of viral IRESs. Most of the known ITAFs are RNA-processing factors with RNA-binding domains (8). There is no report that GADD34 has nucleic acid binding activity. We speculate that GADD34 may be involved in recruiting some key ITAFs, thereby activating various viral IRESs. Goh et al. used mass spectrometry to screen GADD34-interacting proteins and identify GADD34 novel functions (18). We analyzed the results of mass spectrometry and found that GADD34 has potential interactions with various RNA binding proteins or translation initiation related proteins, such as eukaryotic translation initiation factor 3 (eIF3) complex, heterogeneous nuclear ribonucleoproteins (hnRNPs) family, eukaryotic translation elongation Factor 2 (EEF2), and some proteins that have been reported to be involved in IRES, including poly(rC)-binding protein 1 (PCBP1) (59), ATP binding cassette subfamily F member 1 (ABCF1) (60), and DEAD box helicase 3 X-linked (DDX3X) (61). The mechanism by which GADD34 promotes viral IRESs requires further experimentation and study.

The process of virus infection and replication involves complex intracellular molecular interactions between viral and host proteins. Viruses have evolved complex mechanisms to hijack host cell machinery and metabolic pathways, thereby redirecting the flow of resources and energy to facilitate virus replication. This study shows that GADD34 interacts with EV71 3CD, and this interaction contributes to the promotion of virus replication by GADD34. Structurally, GADD34 513 to 631 aa is an intrinsically disordered protein (IDP) region (36). IDP family proteins are variable, flexible, dynamic, and easy to interact with various proteins. It is estimated that 15% to 45% of protein-protein interactions are formed with IDPs (62). We have identified the domain 555 to 567 aa that interacts with EV71 3CD, belonging to the IDP region, which is the structural basis of GADD34 to interact with EV71 3CD. It has been reported that GADD34 483 to 610 aa interacts with Epstein-Barr virus (EBV) protein EBNA3C (63), and GADD34 511 to 674 aa interacts with human T-cell leukemia virus type-1 (HTLV-1) protein HBZ and changes the HBZ localization (64). This evidence completely demonstrated that the C-terminal of GADD34 might be involved in interaction with viral protein. On the other hand, Belov et al. speculated that PV 3CD could interact with some unidentified host membrane proteins (12). Studies have shown that PV 3CD may interact with PIP lipids in the membrane of virus replication (2). Harris et al. proposed that PV 3CD interacts with eukaryotic elongation factor EF-1 alpha (EF-1α) (49). Brunner et al. found that PV 3CD interacts with heterogeneous nuclear ribonucleoprotein (hnRNP) C1/C2 to enhance virus replication (65). Our results provide a new example, ER localization protein GADD34, for the interaction of picornavirus 3CD with host proteins.

Enterovirus 3CD may affect the binding of host protein to viral IRES. PV 3CD respectively binds to the cloverleaf-like structure of 5′-UTR, the *cis*-acting replication RNA element (*cre*) of ORF, and 3'UTR on viral RNA (30, 49). These interactions are very important for viral RNA replication. Gamarnik et al. provided an example of PV 3CD adjusting cellular ITAF combined with PV 5′-UTR. PV 3CD enhanced the binding affinity of PCBP to the cloverleaf of PV 5′-UTR, causing PCBP to dissociate from the stem-loop IV (66). Our study found that EV71 3CD recruited the ER localization protein GADD34 to the EV71 5′-UTR, which breaks the inherent impression that 3CD promotes RNA synthesis but does not participate in the regulation of viral IRES.

In general, our research has broadened the scope of the interaction between the virus and the host cells, providing several new mechanisms for how the virus regulates host gene expression and how the host protein participates in virus replication and provides new insights for screening antiviral drug targets.

## MATERIALS AND METHODS

### Cell culture and virus infection.

HEK293T, HeLa, human RD cells, HT-29 cells, and SH-SY5Y cells were cultured in Dulbecco's modified Eagle's medium (DMEM), Caco-2 cells were cultured in DMEM with non-essential amino acids, Jurkat cells were cultured in RPMI 1640, all supplemented with 10% fetal bovine serum (FBS) and penicillin-streptomycin at 37°C in 5% CO_2_. Virus stocks were produced in RD cells. The infectious clone EV71 Malaysia strain (GenBank: AB469182.1) was linearized, and then the RNA transcribed *in vitro* was transfected into RD cells. According to the manufacturer's protocol, a MEGAscript T7 High Yield transcription kit (Thermo Fisher Scientific) was used to perform *in vitro* transcription. After cell death, the freeze-thaw culture supernatant was harvested and removed the debris to obtain EV71 particles. EV71 was expanded in RD cells for three generations. The viral titers were determined by endpoint titration analysis and expressed as 50% tissue culture infectious dose (TCID_50_). RD cells in 96-well plates were infected. After 6 days, the cells with cytopathic effect (CPE) were counted, and the viral titers were calculated according to the Reed-Muench method. EV71 at the indicated multiplicity of infection (MOI) was incubated with cells in DMEM without FBS for 1 h, and the medium was changed to DMEM with 2% FBS.

### Western blotting.

Cells were harvested and lysed in radio-immunoprecipitation assay (RIPA) buffer containing 50 mM Tris (pH 7.4), 150 mM NaCl, 1% NP-40, 2 mM EDTA, 0.5% sodium deoxycholate, and protease inhibitor cocktail (Roche). The lysate supernatant was mixed with the loading buffer containing 250 mM Tris (pH 6.8), 10% SDS, 50% glycerol, 5% 2-mercaptoethanol, and 0.5% Bromophenol Blue, and boiled at 100°C for 10 min. Samples were run on polyacrylamide gels and transferred to PVDF membranes (GE Healthcare). The PVDF membranes were blocked with 5% nonfat milk in phosphate-buffered saline with Tween 20 (PBST) and then incubated with the primary antibodies. The primary antibodies used were as follows: anti-GADD34 (cat. no. 10449-1-AP, Proteintech), anti-V5 (cat. no. 66007-1-Ig, Proteintech), anti-HA (cat. no. H3663 and H6908, Sigma), anti-Flag (cat. no. F1804, Sigma), anti-Tubulin (cat. no. sc-32293, Santa Cruz), anti-GFP (cat. no. sc-9996, Santa Cruz), anti-P-eIF2α (cat. no. 3398T, Cell Signaling Technology), anti-eIF2α (cat. no. YT1507, ImmunoWay), and anti-VP1 from immunized mice. Membranes were washed three times with PBST and then incubated with the secondary antibodies, anti-rabbit IgG horseradish peroxidase (HRP)-linked antibody (cat. no. 7074S, Cell Signaling Technology) and anti-mouse IgG HRP-linked antibody (cat. no. 7076S, Cell Signaling Technology). Membranes were washed six times with PBST, treated with Immobilon Western Chemiluminescent HRP Substrate (EMD Millipore) for chemiluminescent detection of HRP.

### Reverse transcription and quantitative PCR (RT-qPCR).

Cells were lysed using TRIzol reagent (Invitrogen), and RNA was extracted according to the manufacturer’s protocol. Reverse transcription was performed with the oligo(dT) primer and M-MLV Reverse Transcriptase (Promega). cDNA was analyzed using SYBR green mix (Roche) and the StepOne real-time PCR system (Applied Biosystems). The sequences of the primers used were as follows: *GAPDH*, forward, 5′-AACAGCGACACCCACTCCTC-3′ and reverse, 5′-CATACCAGGAAATGAGCTTGACAA-3′; *GADD34*, forward, 5′-TCCGAGTGGCCATCTATGTA-3′ and reverse, 5′-AGGGTCCGGATCATGAGTAG-3′; EV71, forward, 5′-GCAGCCCAGAAGAACTTTAC-3′ and reverse, 5′-ATTTCAGCAGCTTGAAGCGC-3′. Each sample contains three technical replicates. Fold change of RNA was calculated using the threshold cycle (ΔΔ*C_T_*) method.

### Plasmids and transfection.

The EV71 infectious clone, pSVA-EV71, was a gift from Zhiyong Lou (Tsinghua University). The Flag-tagged vector pCE-puro-3×Flag was a gift from Akio Kihara (Hokkaido University). pcDNA3.1-IRES-2A-V5-HisA was a gift from Shih-Yen Lo (Tzu Chi University). GFP-tagged EV71 series of expressed proteins were gifts from Fei Guo (Chinese Academy of Medical Sciences and Peking Union Medical College). EV71 3A, 3AB, 3C, 3D, and 3CD were PCR amplified to add BamHI in the upstream and *Not*I in the downstream using pSVA-EV71 as a template, and then inserted into pCE-puro-3×Flag vector. The *GADD34* 5′-UTR, GADD34 CDS, and hnRNP K CDS were amplified from human cDNA and inserted into vectors. The bicistronic reporter vector pRF was a gift from Anne E. Willis (University of Cambridge). The hairpin sequences were annealed and inserted into pRF to obtain pRHF. For the first annealing and insertion, pRF was digested with *Spe*I and *Pvu*II, the primers used for the annealing were: forward, 5′-CTAGTAGATCTGGTACCGAGCTCCCCGGGCTGCAGCAG-3′ and reverse, 5′-CTGCTGCAGCCCGGGGAGCTCGGTACCAGATCTA-3′. For the second annealing and insertion, the product of the first round was digested with *Pst*I and *Pvu*II, the primers used for the annealing were: forward, 5′-GCCCGGGGACCTCGGTACCAGATCTCAG-3′ and reverse, 5′-CTGAGATCTGGTACCGAGGTCCCCGGGCTGCA-3′. EV71 5′-UTR was amplified from pSVA-EV71. EV71 subgenomic replicon (EV71-Luc) was a gift from Wenhui Li (National Institute of Biological Sciences, Beijing) (40). The IRES of HIV-1, MMTV, and HCV were gifts from Marcelo López-Lastra (Pontificia Universidad Católica de Chile). The IRES of PV and FMDV were synthesized by the company. All point mutants were obtained by QuikChange site-directed mutagenesis method. Cells were transfected with polyethyleneimine (PEI) (Polysciences) or Lipofectamine 3000 (Thermo Fisher Scientific) following the manufacturer’s instructions.

### GADD34 knockdown.

GADD34 in Jurkat and HeLa cells was knocked down by shRNA. Double-stranded oligonucleotides corresponding to the target sequences were inserted into pSIREN-RetroQ. The primer sequences were: forward, 5′-GATCCGGACGATGAAGAAGCTGTATTCAAGAGATACAGCTTCTTCATCGTCCTTTTTTG-3′, and reverse, 5′-AATTCAAAAAAGGACGATGAAGAAGCTGTATCTCTTGAATACAGCTTCTTCATCGTCCG-3′. The pMLV-Gag-Pol, pVSV-G, and pSIREN-RetroQ-shGADD34 were cotransfected into HEK293T cells to prepare lentiviral particles. The collected supernatant was used as the shRNA pseudovirus stock, and it was directly mixed with Jurkat or HeLa cells to knock down GADD34. GADD34 in RD cells was knocked down by transfected siRNA. The sequences used to synthesize siRNA were: siRNA#1, sense, 5′-GGACACUGCAAGGUUCUGATT-3′, and antisense, 5′-UCAGAACCUUGCAGUGUCCTT-3′; siRNA#2, sense, 5′-CGAAGAAACUGGGAAAGGATT-3′, and antisense, 5′-UCCUUUCCCAGUUUCUUCGTT-3′. The siRNA#1 and #2 were transfected into RD cells in a 1:1 mixing manner using Lipofectamine 3000 (Thermo Fisher Scientific).

### Reporter vectors and luciferase assay.

Cells were harvested 48 h after the reporter plasmids were transfected to detect the luciferase activity. *Renilla* luciferase (RL) and firefly luciferase (FL) dual-luciferase activities were measured using a Dual-Luciferase Reporter Assay System (Promega), FL single-luciferase activity was measured using a Luciferase Assay System (Promega), and β-galactosidase (β-gal) activity was measured using ONPG (Sigma) according to the manufacturer’s protocol. For the pRHF dual-luciferase series, FL activity was normalized to RL activity. For the FL single-luciferase series and EV71-Luc, FL activity was normalized to β-gal activity. Signals were measured using a luminometer.

### Immunofluorescence.

HeLa cells were plated on coverslips and harvested at 48 h posttransfection. After being washed twice with phosphate-buffered saline (PBS), cells were fixed with 4% paraformaldehyde diluted by PBS and permeabilized using 0.1% Triton X-100. Then fixed cells were washed five times with PBS and incubated in blocking solution for 2 h. After washing off the blocking solution, cells were incubated with anti-HA (1:200; cat. no. H6908, Sigma) and anti-Flag (1:100; cat. no. F1804, Sigma) diluted in PBS containing 0.1% bovine serum albumin (BSA) for 2 h. Cells were washed three times with PBST and incubated with fluorescein (FITC) goat anti-mouse IgG (1:500; cat. no. 115-095-003, Jackson ImmunoResearch) and rhodamine (TRITC) goat anti-rabbit IgG (1:500; cat. no. 111-025-003, Jackson ImmunoResearch) for 1 h. Following four washes with PBS, cells were counterstained with 4′,6-diamidino-2-phenylindole (DAPI, 1:2,000), and mounted on slides with Antifade Mounting Medium (Beyotime) for confocal microscopy analysis. The images were captured by Leica TCS SP5 confocal system.

### Coimmunoprecipitation.

HEK293T cells were plated in 10-cm dish and harvested 48 h after transfection. Cells were lysed with RIPA buffer containing protease inhibitor cocktail (Roche) on ice. Lysates were incubated with mouse monoclonal anti-HA antibody (cat. no. H6908, Sigma) for 3 h, then incubated with protein A-agarose beads (Santa Cruz) for 2 h, both at 4°C on a rotator. The immune mixture was washed six times with RIPA to remove the nonspecific binding, and then added loading buffer and boiled at 100°C for Western blotting.

### RNA-protein immunoprecipitation.

Cells were processed in the same way as co-immunoprecipitation. Lysates were divided into two equal parts: one part was incubated with anti-HA (cat. no. H6908, Sigma) or anti-Flag (cat. no. F1804, Sigma), and the other part was incubated with normal IgG, for 2 h at 4°C on a rotator. Then all samples were incubated with protein A-agarose beads (Santa Cruz) for 1 h at 4°C on a rotator. After being washed five times with RIPA, immunoprecipitated RNA-protein complexes were digested with Proteinase K (TransGen), and RNA was extracted using a RNeasy minikit (Qiagen). The reverse transcription method was the same as above. cDNA or ddH_2_O was amplified with EV71 5′-UTR specific primers (forward, 5′-GCCCCTGAATGCGGCTAATC-3′, and reverse, 5′-CATGTTTGACTGTATTGAGAG-3′) and imaged by agarose gel electrophoresis.

### Data availability.

The sequencing data were deposited at the Gene Expression Omnibus (GEO) repository. The accession number is GSE103308.
