# Supplementary material for: Enterovirus 71 Activates GADD34 via Precursor 3CD to Promote IRES-Mediated Viral Translation
Source: Microbiol Spectr. 2022 Jan 5;10(1):e01388-21. doi: 10.1128/spectrum.01388-21 (PMC8729766; doi:10.1128/spectrum.01388-21)
Supplement: SUPPLEMENTAL FILE 1 — Supplemental material. Download Spectrum01388-21_Supplemental_Material.pdf, PDF file, 0.5 MB [file spectrum01388-21_supplemental_material.pdf]

TABLE S1 Genes that were differentially up-regulated at 3.5 h and 6.25 h after EV71 infection of RD cells

3.5 h mRNA		3.5 h RPF		6.25 h mRNA		6.25 h RPF	
Gene	Log_2(FC)	Gene	Log_2(FC)	Gene	Log_2(FC)	Gene	Log_2(FC)
EGR1	4.0180	FOS	4.2505	EGR1	4.6393	SNORD114-1	5.1884
RNY4P31	3.9408	CYR61	2.9791	FOS	4.6134	FOS	4.0887
FOS	3.5970	GADD45B	2.6094	CYR61	4.2126	GADD34	3.5593
ZNF844	2.8729	ADM	2.4581	ZNF844	3.0250	GADD45B	3.3425
CYR61	2.6131	PIM1	2.1538	NUAK2	2.9318	ZNF844	3.1631
NUAK2	2.1640	EGR1	1.9027	PIM1	2.7335	EGR1	3.0372
PIM1	1.7948	ZC3H12A	1.8756	GADD45B	2.4872	RNY4P31	3.0270
RNU5E-4P	1.7708	GADD34	1.8652	ADM	2.2611	CYR61	3.0094
NFKBIZ	1.7573	ZNF367	1.7226	HES1	2.1286	RNU5E-4P	2.9654
GADD45B	1.7511	NUAK2	1.6854	ZNF367	2.0930	ATF3	2.9454
HES1	1.6819	HES1	1.6062	ZNF44	2.0162	ADM	2.8911
ZC3HAV1	1.4897	KLF4	1.5661	RNU5E-4P	1.9257	CLK1	2.7172
ZNF44	1.4102	ATF3	1.4703	ZC3HAV1	1.8775	KLF4	2.5942
ZC3H12A	1.3388	CLK1	1.4306	KLF4	1.7826	ZNF367	2.3882
ADM	1.2117	ZNF844	1.4298	ZC3H12A	1.7717	PIM1	2.2983
ZNF367	1.1985	ZC3HAV1	1.4226	CLK1	1.6727	HES1	2.1679
ATF3	1.1557	NFKBIZ	1.2879	ATF3	1.6478	NFKBIZ	2.0609
KLF4	1.1260	SNORD114-1	1.2705	GADD34	1.6396	NUAK2	1.8661
SNORD114-1	1.1031	RNU5E-4P	1.2041	NFKBIZ	1.6128	ZC3H12A	1.7976
GADD34	1.0786	RNY4P31	1.1335	RNY4P31	1.2453	ZC3HAV1	1.5734
CLK1	1.0336	ZNF44	1.1125	SNORD114-1	1.0365	ZNF44	1.4279

TABLE S2 Differentially up-regulated genes with TE (RPF/mRNA) >1

3.5 h		6.25 h	
Gene	TE	Gene	TE
ADM	2.0286	SNORD114-1	5.0055
GADD34	1.7293	GADD34	2.1708
GADD45B	1.4901	ATF3	1.7875
ZNF367	1.4374	CLK1	1.6244
ZC3H12A	1.4010	KLF4	1.4553
KLF4	1.3908	GADD45B	1.3438
CLK1	1.3841	ADM	1.2786
ATF3	1.2723	ZNF367	1.1410
SNORD114-1	1.1517	ZC3H12A	1.0146

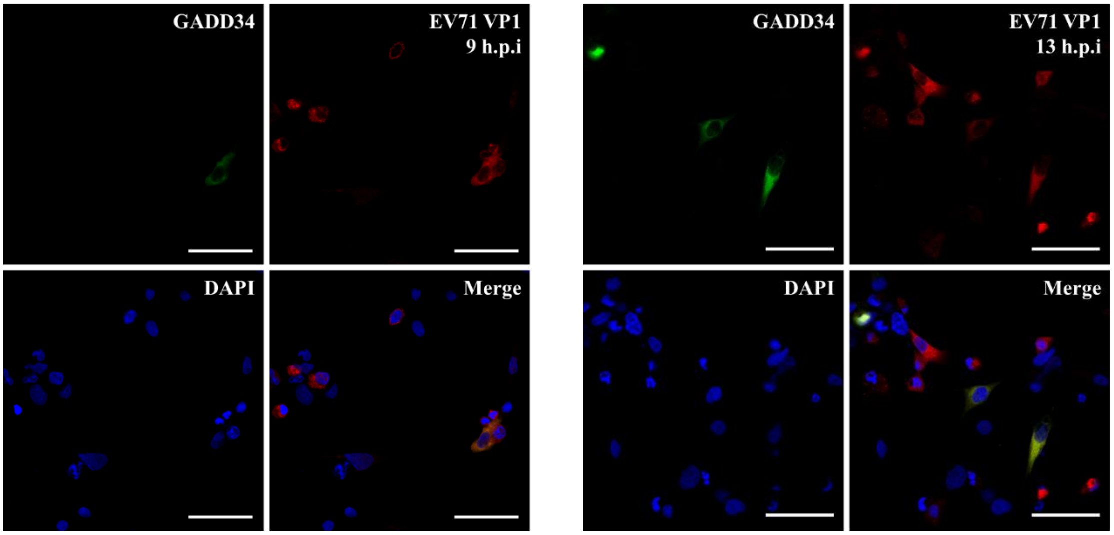

FIG S1 GADD34 is up-regulated in RD cells infected with EV71. RD cells were infected with 5 MOI EV71 for 9 h and 13 h. RD cells were fixed, and the protein localization was detected by immunofluorescence. Bars represent 50 µm.

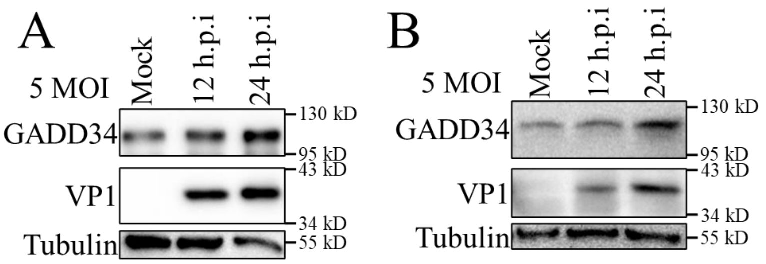

FIG S2 GADD34 is up-regulated by EV71 infection in Caco-2 cells and SH-SY5Y cells. SH-SY5Y cells (A) and Caco-2 cells (B) were infected with 5 MOI EV71, and the cells were harvested at 12 h and 24 h after infection. GADD34 was detected by Western blotting.

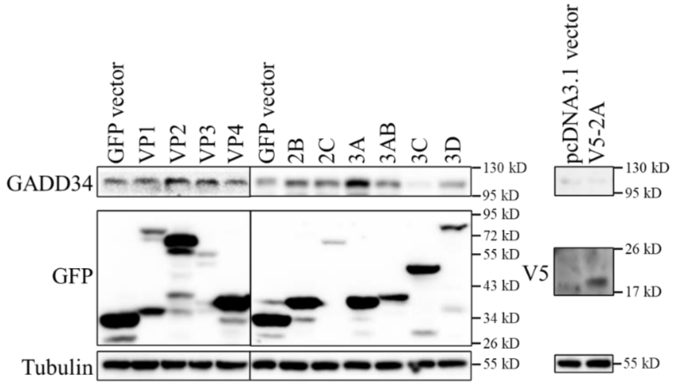

FIG S3 Screen the EV71 components that activate GADD34 protein. Plasmids of EV71 GFP-tag proteins and V5-tag 2A were transfected into HeLa cells, and GADD34 protein expression was detected by western blotting after 48 h.

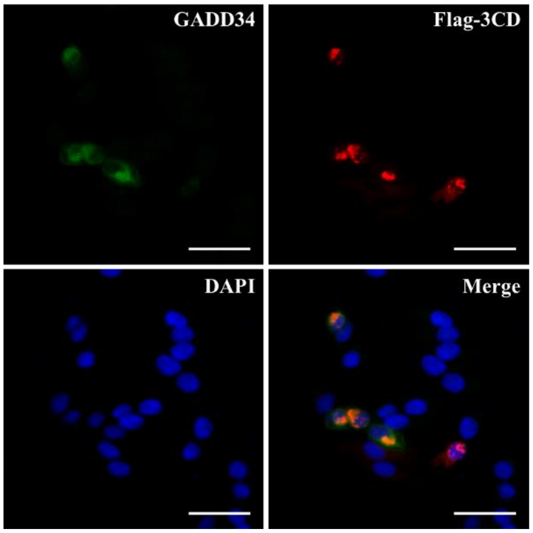

FIG S4 GADD34 is up-regulated by EV71 3CD. Flag-3CD was transfected into HeLa cells. 48 h later, cells were fixed, and the protein localization was detected by immunofluorescence. Bars represent 50 μm.

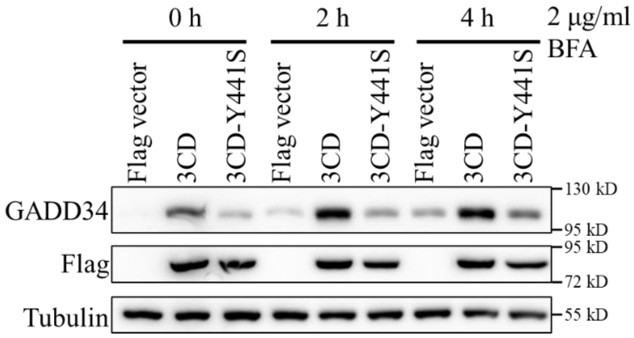

FIG S5 GADD34 is induced by EV71 3CD independent of Arf1. HeLa cells were transfected with pCE-puro-3×Flag-3CD and 3CD-Y441S. After 24 h, cells were treated with 2μg/ml Brefeldin A (BFA) for 0, 2, and 4 h and then harvested. Proteins were detected by Western blotting.

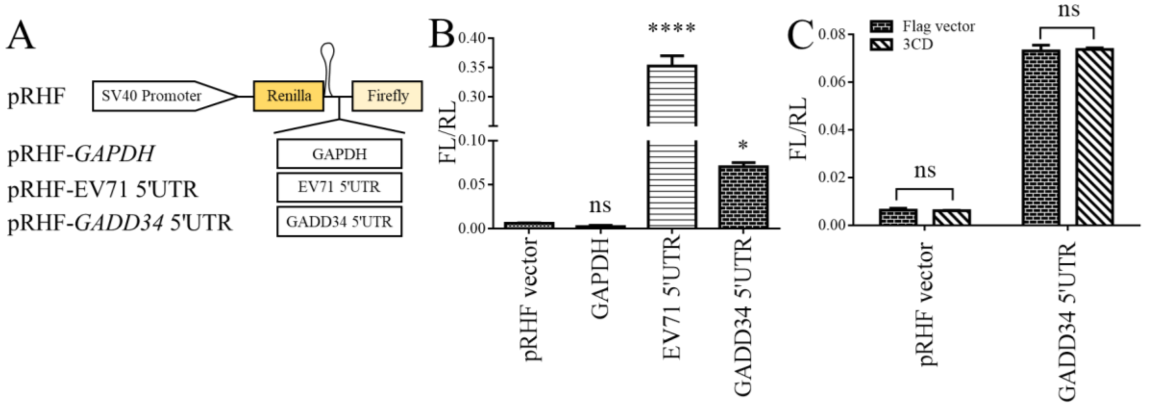

FIG S6 EV71 3CD does not affect the IRES activity of *GADD34* 5'-UTR. (A) *GADD34* 5'-UTR, positive control EV71 5'-UTR, and negative control *GAPDH* 951-1250 bp were constructed into dual luciferase reporter vector pRHF. (B) The plasmids constructed in (A) were transfected into HeLa cells, then FL and RL activities were detected after 48 h. (C) HeLa cells were co-transfected with EV71 3CD and pRHF or pRHF-*GADD34* 5'-UTR, FL and RL activities were detected 48 h after transfection. Values were the means plus standard errors of the means (SEM) (error bars) from two individual experiments. Values were statistically evaluated using a one-way ANOVA (B) or a two-tailed unpaired *t* test (C). *, *P* < 0.05; ****, P < 0.0001; ns, not significant.

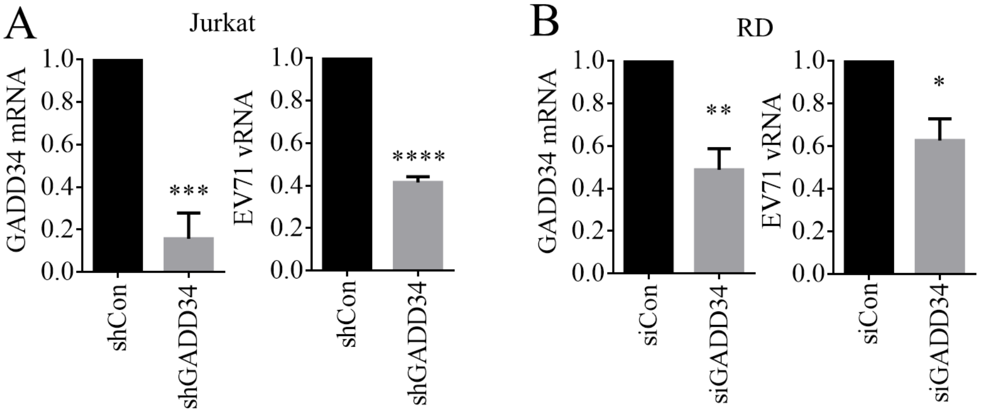

FIG S7 Knockdown of GADD34 inhibits EV71 replication. (A) The suspended Jurkat cells were infected with pSIREN-RetroQ-shGADD34 or shControl packaged lentivirus. Two days later, cells were infected with 0.1 MOI EV71 for 12 h. The harvested cells were used to detect the knockdown effect of GADD34 and the vRNA of EV71 by RT-qPCR. (B) RD cells were transfected with siCon or siGADD34. 24 h after transfection, cells were infected

with 1 MOI EV71 for 8 h. In harvested cells, RNA was detected by RT-qPCR. Values were the means plus standard errors of the means (SEM) (error bars) from three individual experiments. Values were statistically evaluated using a two-tailed unpaired *t* test. *, P < 0.05; **, P < 0.01; ***, P < 0.001; ****, P < 0.0001.

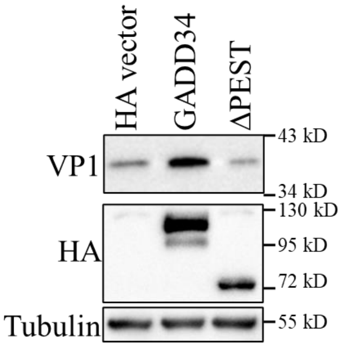

FIG S8 Deleting the PEST of GADD34 does not promote EV71 replication. RD cells were transfected with HA-GADD34 or HA-GADD34-ΔPEST. Cells were infected with 1 MOI EV71 24 h after transfection. Then cells were harvested 8 h after infection to detect proteins by Western blotting.

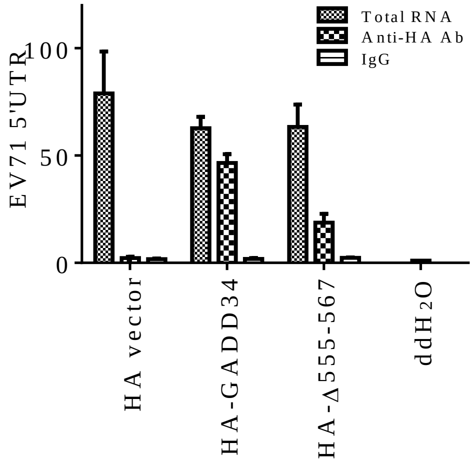

FIG S9 Deletion of 555-567 aa of GADD34 weakens the recruitment to EV71 5'-UTR. HA

vector, HA-GADD34, or HA-GADD34-Δ555-567 and pRHF-EV71 5'-UTR and 3×Flag-3CD were co-transfected into HEK293T cells. After 48 h, cells were lysed for RNA-protein immunoprecipitation (RIP). Proteins were precipitated by anti-HA antibody and beads, and the cDNA reverse-transcribed from the pulled RNA was quantified by RT-qPCR. Values were the means plus standard errors of the means (SEM) (error bars) from two individual experiments.
